# Complexity and evidence in health sector decision-making: lessons from tuberculosis infection prevention in South Africa

**DOI:** 10.1093/heapol/czac059

**Published:** 2022-07-29

**Authors:** Shehani Perera, Justin Parkhurst, Karin Diaconu, Fiammetta Bozzani, Anna Vassall, Alison Grant, Karina Kielmann

**Affiliations:** Division of Social and Behavioural Sciences, School of Public Health and Family Medicine, University of Cape Town, Falmouth Road, Observatory 7925, Cape Town, South Africa; Department of Health Policy, London School of Economics and Political Science, Houghton Street, London WC2A 2AE, England, United Kingdom; Institute for Global Health and Development, School of Health Sciences, Queen Margaret University, Musselburgh EH21 6UU, Edinburgh, UK; Department of Global Health and Development, London School of Hygiene and Tropical Medicine, Keppel Street, London WC1E 7HT, England, UK; Centre for Health Economics in London, London School of Hygiene & Tropical Medicine, Keppel Street, London WC1E 7HT, England, UK; TB Centre, London School of Hygiene & Tropical Medicine, Keppel St, London WC1E 7HT, England, UK; Africa Health Research Institute, School of Laboratory Medicine & Medical Sciences, College of Health Sciences, University of KwaZulu-Natal, 719 Umbilo Road, Durban 4013, South Africa; School of Public Health, University of the Witwatersrand, 27 St Andrews Road, Parktown 2193, Johannesburg, South Africa

**Keywords:** System dynamics modelling, health policy, evidence-based research, tuberculosis, South Africa

## Abstract

To better understand and plan health systems featuring multiple levels and complex causal elements, there have been increasing attempts to incorporate tools arising from complexity science to inform decisions. The utilization of new planning approaches can have important implications for the types of evidence that inform health policymaking and the mechanisms through which they do so. This paper presents an empirical analysis of the application of one such tool—system dynamics modelling (SDM)—within a tuberculosis control programme in South Africa in order to explore how SDM was utilized, and to reflect on the implications for evidence-informed health policymaking. We observed group model building workshops that served to develop the SDM process and undertook 19 qualitative interviews with policymakers and practitioners who partook in these workshops. We analysed the relationship between the SDM process and the use of evidence for policymaking through four conceptual perspectives: (1) a rationalist knowledge-translation view that considers how previously-generated research can be taken up into policy; (2) a programmatic approach that considers existing goals and tasks of decision-makers, and how evidence might address them; (3) a social constructivist lens exploring how the process of using an evidentiary planning tool like SDM can shape the understanding of problems and their solutions; and (4) a normative perspective that recognizes that stakeholders may have different priorities, and thus considers which groups are included and represented in the process. Each perspective can provide useful insights into the SDM process and the political nature of evidence use. In particular, SDM can provide technical information to solve problems, potentially leave out other concerns and influence how problems are conceptualized by formalizing the boundaries of the policy problem and delineating particular solution sets. Undertaking the process further involves choices on stakeholder inclusion affecting whose interests may be served as evidence to inform decisions.

Key messages

- System dynamics modelling (SDM) is increasingly being used to inform health policy and systems decision-making
- The formal implementation of SDM can serve as a knowledge translation and evidence provision tool for policymaking
- There are multiple ways to consider how SDM might affect evidence-informed policymaking, including: as a direct provision of information, as a way to identify needed evidence, or as a process by which possible problems and their solutions can be formalized.

## Introduction

Planning health system activities can be a challenging process, requiring information about a range of variables and relationships that can shape service delivery and ultimately clinical outcomes for populations. To assist health sector decision-makers with such issues, there have been increasing efforts to engage with complexity sciences and the tools this field offers (Carey *et al.*, 2015; Chang *et al.*, 2017). System dynamics modelling (SDM) is one specific tool developed to analyse complex systems, identify problems and evaluate potential solutions (Forrester, 1969). As a tool that combines components of cybernetics, organizational theory, information technology and control engineering, it can help decision-makers identify and apply a range of information to address complex and dynamic policy problems (Eker *et al.*, 2017). SDM has increasingly been applied in the health sector to help inform and guide local health policy and planning efforts (Atkinson *et al.*, 2015; Darabi & Hosseinichimei, 2020). However, the application of such a tool can also fundamentally shape the ways that various forms of evidence are identified, brought to bear upon and applied to inform policy decisions.

There has been some work reviewing the application of SDM to characterize health systems (Chang *et al.*, 2017) or to support health policy (Atkinson *et al.*, 2015), as well as broader discussions of its use and implications within public policymaking (see Stave, 2002; Goertz, 2006; Teisman and Klijn, 2008). However, there has been limited research on how the use of SDM relates to evidence use in public policy making spaces (for one conceptual discussion, see Malbon and Parkhurst (2022), arising from this research project). This can be important, however, given that there are significant political considerations involved in the construction, selection and application of evidence within public policy spaces more broadly (Parkhurst, 2017). In this paper, we present an empirical analysis that reflects on the programmatic implications of the application of SDM (in particular the group model building (GMB) approach), within an infection control project for tuberculosis (TB) in South Africa to explore these considerations. While TB serves as point of entry, the paper uses this case study to critically reflect on how the application of this complexity-informed planning tool relates to key elements of evidence use within health policymaking.

### Empirical and conceptual background

#### Tuberculosis infection prevention and control

TB is a significant global public health concern, with a total of 1.4 million people dying from the disease in 2019 (World Health Organization, 2020). According to the World Health Organization (WHO), South Africa has one of the highest burdens of drug-resistant TB (DR-TB), with approximately 14 000 new cases diagnosed in 2019 (World Health Organization, 2020). Ending the epidemic by 2030 is one of the health targets of the Sustainable Development Goals, and the *End TB Strategy* outlines three strategic pillars to help achieve this—one of which is integrated patient-centred care and prevention, encompassing TB infection prevention and control (IPC) (END TB Strategy, 2014; World Health Organization, 2014).

IPC measures seek to prevent patients and healthcare workers from avoidable infection (World Health Organization, 2021). For TB IPC, there is particular emphasis on the ‘combination of measures designed to minimize the risk of TB transmission within populations’ (World Health Organization, 2019). In South Africa, weak TB IPC implementation has been linked to staff shortages, long waiting times, lack of IPC training support, poor occupational health and safety practices and conflicting policy guidelines (Zinatsa *et al.*, 2018; van der Westhuizen *et al.*, 2019; Zwama *et al.*, 2021).

Within SDM literature, this set of problems could be referred to as ‘messy problems’ given their ‘tangled’ nature and lack of straightforward definition or solution (Vennix, 1995, p. 336; Dawes *et al*., 2009, cited in Black, 2013, p. 71). For instance, in the case of TB IPC, long waiting times themselves can be a direct risk factor for the spread of an infectious disease in a clinic setting, as can sub-optimal health and safety practices. Yet both can be affected by staff shortages—with remaining staff making tradeoffs between undertaking IPC training, working to improve clinic settings and seeing patients in a timely manner. Vennix (1995) has argued that decision-making for these types of problems can be facilitated by the GMB approach of SDM, as this can promote insight, establish a shared understanding of the problem, function as an effective communication tool and create consensus among various stakeholders.

#### Group model building as an approach to system dynamics modelling

SDM is recognized as having a clearly defined methodology for problem-solving and has therefore become increasingly applied to understand policy problems and guide policy decision-making in several fields (Stave, 2002; Carey *et al.*, 2015; McLucas and Elsawah, 2020). The methodology suits addressing issues of complexity where computer simulation models of accumulation (people, material, financial assets, information etc.) and feedback are tested systematically to identify policy solutions (Homer and Hirsch, 2006). However, creating a ‘system’ view of the problem is not easily accomplished by any one stakeholder as each is invested in their own knowledge and cannot effortlessly discern others’ objectives and tasks (Carlile, 2002, cited in Luna-Reyes *et al.*, 2019, p. 5). A facilitated approach to systems modelling such as GMB can therefore create a common context for interdisciplinary work and has proven useful with groups of researchers engaged in theory-building and -testing (Rouwette *et al.*, 2002; Luna-Reyes *et al.*, 2019).

At a group level, GMB’s goals include: (1) learning by helping people gain more insight into the structure and behaviour of the system; (2) creating agreements/consensus about policy decisions; and (3) generating commitment with a decision (Andersen *et al.*, 1998). At the organizational level, its goals are to do things differently (system process change) and/or assess whether stakeholders are impacted differently (systems outcome change) (Cavaleri & Sterman, 1997, cited in Andersen *et al.*, 1998). While variations of GMB processes exist, the SDM GMB process typically consists of a problem definition meeting, group modelling meeting, formal model formulation by the modelling team, model review, final conceptual modelling and presentation of project results and implementations (Vennix, 1995; Richardson and Andersen, 2019). GMBs are conducted using scripts which are ‘behavioural descriptions of pieces of a facilitated group exercise that move a group forward in a systems thinking intervention’ (Andersen *et al.*, 1998, p. 193; Richardson and Andersen, 2019). These scripts maintain focus on the key variables of interest and help to develop a shared understanding of the problem (Richardson and Andersen, 2019).

Black (2013) highlights how GMB functions to create these socially constructed agreements among individuals holding different values, understandings and goals and makes the argument that visual representations, ‘boundary objects’, can greatly improve GMB outcomes (Black, 2013). Boundary objects, simply put, are representations in the form of ‘a diagram, sketch, sparse text, or prototype that helps individuals collaborate effectively across some boundary’ during the GMB intervention (Black, 2013, p. 7). Luna-Reyes *et al*. (2019, p. 2) similarly explain that: ‘Simulation models, as well as other artifacts used during the [group] modeling process, work as boundary objects useful to facilitate conversations among researchers of different disciplines, uncover insights, and build consensus on causal connections and actionable insights’.

#### Considerations in relation to evidence use

In this section, we outline four distinct conceptual perspectives which exist in the broader literature theorizing the use of evidence for policymaking. Each of these will be taken to reflect on SDM’s use in relation to evidence and policymaking within the GMB intervention in our specific case study. Table 1 summarizes the key questions arising from each of these concerns (detailed below) which are then applied to structure the subsequent results sections. The first perspective considered is explicitly rationalist. By formalizing a process through which problems can be better understood, SDM can provide an opportunity for decision-makers to identify which pieces of evidence may be useful to solve problems. This may be particularly relevant when the modelling process is linked to academic research that has generated evidence explicitly aiming to be policy relevant. In this way, the application of SDM derived from research can be seen as a tool utilizing a knowledge translation (KT) process. KT work often begins by asking questions about a given body of evidence to try to understand why or how it was taken up or used within decision-making (Contandriopoulos *et al.*, 2010; Oliver *et al.*, 2014a). In doing so, processes of evidence utilization are seen to fill what Kitson *et al*. (2018, p. 232) have described as ‘translation gaps’ in knowledge production—wherein ‘KT appears to be slow or incomplete’. The KT view of evidence utilization, however, has been critiqued by policy scholars who have argued it is based on a naïve reduction of policymaking to simple evaluations of technical evidence (Russell *et al.*, 2008). Rather, it is recognized that decision-makers can have multiple, often competing, policy needs, and thus evidence ‘use’ can mean many things (Nutley *et al.*, 2007; Oliver *et al.*, 2014b; Cairney, 2016).

**Table 1.** Perspectives and key questions

Perspective	Key questions
Knowledge translation	Does SDM facilitate application or identification of useful evidence generated by researchers?
Programmatic perspective of evidence use	What are the tasks and goals of decision-makers? How (and how well) does SDM provide evidence to serve those tasks and goals?
Post-structuralist concerns for problem and solution set construction	How does the use of evidence within the SDM process specify the bounds and constructions of decision-making concepts?
Representation and prioritization of social concerns	Which stakeholders are involved in the SDM process and how?

A second perspective is to take a so-called ‘programmatic approach’ to evidence utilization (as developed by Parkhurst *et al.*, 2020) which starts by clarifying the tasks, roles and goals that bureaucratic actors pursue, so as to consider the sources, forms and targets of evidence that decision-makers find to be appropriate in relation to their institutionalized goals. While this approach is still concerned with evidence use from a problem-solving perspective, it is more critical in its recognition of the multiple goals and needs of decision-makers. As such, it allows consideration of how and when modelling processes provide evidence that is relevant to a range of tasks, or whether there may be gaps in needed information.

A third approach reflects on evidence use from a constructivist orientation. As Lancaster (2014, p. 948) explains, what is considered ‘policy-relevant knowledge’ is not ‘a stable concept but rather one which is constructed through the policy process, and, through a process of validation, is rendered useful’. Post-structuralist policy scholars in particular have focused inquiries into how policy issues are constructed to critically reflect on which ideas and interests end up built into, or left out of, particular problematizations (Bacchi and Goodwin, 2016). From this perspective, the tools used to inform policymaking can be analysed in relation to how they serve to develop particular issue constructions, with Lascoumes and le Gales (2007, p. 1) arguing that public policy instruments and tools have productive effects to ‘structure public policy according to their own logic’. Applied to SDM, this allows consideration of how the process of developing and applying SDM—including the utilization of evidence within this process—serves to construct problems and solutions as policy-relevant.

The final perspective on evidence use in policymaking, however, focuses more explicitly on normative concerns over stakeholder representation in shaping what is valued in policymaking. In an applied policy sector such as health, issues of problem construction are not merely theoretical. Rather, there are important implications in relation to whose interests are articulated and which social concerns are prioritized (Hoppe, 2011). The implication of this insight is that it can be important to specifically look at how planning tools marshalling evidence include or represent different stakeholders, as the inclusion or exclusion of particular stakeholders may have implications for which interests and ideas are represented in the discussions and final decisions.

Each of these perspectives—rationalist concerns over knowledge use; programmatic appropriateness to multiple goals; post-structuralist interest in problem construction; and representation of stakeholders—provides a separate but complimentary lens by which to reflect on the use of evidence for policy. They each also raise different, yet important questions about how SDM operates in relation to evidence, as summarized in Table 1, and are used to frame the analysis of our empirical case study.

## Methods

This paper explores these issues through the analysis of an application of SDM to help inform IPC for DR-TB in South Africa. Specifically, we present findings from an ancillary study affiliated with the Umoya Omuhle (UO) project (translation ‘good air’ from isiZulu) which was undertaken from 2017 to 2020 with an aim to identify the diverse drivers of nosocomial transmission of DR-TB in South African primary care facilities. As TB IPC represents a complex intervention that is delivered within a dynamic context shaped by myriad factors such as policy guidelines, organizational culture and resource availability, the UO project used a ‘whole systems approach’ to examine social, biological and infrastructure-related transmission dynamics in two South African provinces to inform health systems interventions (see Kielmann *et al.*, 2020). In addition to data collection, the UO project used the GMB approach to generate a visual diagram of the relationships within and across factors influencing the implementation of TB IPC. This exercise—undertaken through facilitated workshops with local decision-makers, health practitioners and patient advocates—served as a conduit through which evidence and research findings could inform policy deliberations around optimal interventions to improve TB IPC.

### *The* Umoya Omuhle *project and its GMB exercise*

To inform TB control planning, UO researchers collected a set of data at clinics in two South African provinces (Western Cape and KwaZulu-Natal). This included data on clinic infrastructure, space and resources; on the policies, norms and values governing processes of care and IPC for staff and patients; and on the prevalence of TB among clinic attendees. Once primary data had been collected, UO facilitated a GMB exercise consisting of two workshops with high-level national and provincial policymakers, public health professionals and patient advocates engaged in DR-TB service design and delivery. An initial list of participants was drawn up by researchers to capture a range of diverse insights into the complex problem of TB IPC in South Africa. To capture policy insights, participants responsible for designing clinics and infrastructure and TB- and HIV-related policymakers were purposively targeted. To capture clinic-based perspectives, nurses, district health managers and occupational health representatives were invited. Convenience sampling was used where participants from the original list could not attend. Overall, nine policymakers, 15 practitioners and nine researchers participated in each of the two workshops. While participants were informed of the study’s purpose and research activities before the GMB workshops, most were not familiar with the SDM process.

The first workshop held in person in August 2019 focused on the development of causal loop diagrams (CLDs). On Day 1, policy- and decision-makers at the national and provincial level focused on identifying distal and broader macro-level health system and policy influences on TB prevention and care. As primary data was not distributed ahead of the workshops, a variable elicitation exercise was also conducted to identify key events and factors influencing TB burden in South Africa. On Day 2, practitioners focused on identifying proximal factors related to TB care delivery and nosocomial transmission which could be linked to Day 1’s identified factors. For each participant group, a researcher and expert modeller then guided the development of an initial CLD and assisted with the identification of points of fragility and intervention (areas that were fragile/weak and, among these, areas that were amenable to change based on the interventions’ expected effectiveness and feasibility).

The second workshop was conducted virtually due to COVID-19 restrictions in October 2020 and reviewed both the model generated as well as the preliminary mathematical and economic modelling results. UO researchers were embedded in the GMB to be able to critically feed in insights from the collected data. Participants then discussed core intervention mechanisms and researchers further elaborated the mechanisms and, at follow-up calls, repeatedly checked in with participants to validate.

#### Data collection and analysis for the sub-study

For this paper, a sub-study was conducted around the GMB workshops. Individual interviews with participants involved in these workshops were undertaken face to face after the first workshop, and via phone/video call after the second. We interviewed eight policymakers and three practitioners after the first workshop and five policymakers and three practitioners after the second workshop (see Table 2). As many of the participants were also involved in COVID-19 service delivery at this time, further interviews could not be secured. During these interviews, participants were asked to reflect on how the SDM process related to their tasks, goals and interests, what they considered the most ‘appropriate’ evidence in relation to their work—including what information they might require, which aspects made this information useful and which actors were most important in providing evidence to them. It is worth noting that the term ‘evidence’ itself can mean many things depending on the audience. While utilization of policy-relevant research findings (such as those produced by the broader UO project) would be one key form of evidence to consider, in our interviews and the results below we include other related used terms including ‘data’ and ‘information’. This both reflects the language used by participants and delineates a slightly broader range of policy-relevant factual information that was seen as important to include beyond findings from the research project alone.

**Table 2.** Participant information

Type of participant	Definition	Institution	No. of interview participants from First Workshop	No. of interview participants from Second Workshop
Policymaker	Participants involved in the conceptualization and development of guidelines and programmes seeking to improve TB IPC in South Africa	National Department of Health (NDOH); Provincial Department of Health (PDOH); Professional Councils; think tanks	8	5
Practitioner	Participants actively engaged in provision of TB-related services	Primary health care facilities; hospitals	3	3

Observational notes from the first GMB workshop, video recordings of the second workshop and post-workshop interview data were analysed using thematic analysis. After interviews had been transcribed, they were imported into DEDOOSE software for coding by SP and JP. Interviews were coded using a framework based on programmatic and constructivist concepts as detailed in the conceptualization section above and were refined throughout the data analysis stage. An iterative approach was taken to identify the main themes and sub-themes as they link back to the four perspectives, with the assistance of co-authors (KD, FB, AV, AG and KK) (shown in Table 3). Relevant quotes and excerpts were then exported from DEDOOSE and placed under the thematic categories to facilitate analysis and presentation of results (see the following section).

**Table 3.** Main themes, sub-themes and link to four perspectives

Perspective	Main theme	Sub-theme
Knowledge translation	SDM as a knowledge translation tool	*Scoping problems and solutions* *Knowledge gap identification*
Programmatic perspective of evidence use	Programmatic tasks, goals and relevant evidence	*Goals excluded from consideration*
Post-structuralist concerns for problem and solution set construction	Research objectives as setting the boundaries for evidence use	*SDM concepts established and reflected in discourse* *How established concepts shape evidence use*
Representation and prioritization of social concerns	Participation and representation	*Who was included, when or for what*

## Results

In this section, we present the key findings of this sub-study according to the conceptual concerns detailed above. First, in line with understanding SDM as a KT tool, we consider how its use is linked to evidence use from a rationalist perspective. In relation to the programmatic perspective on SDM, the second section explores participants’ views on what they considered relevant evidence in relation to programmatic tasks and goals. Third, in line with the post-structuralist concerns with problem-structuring, we explore how the research objectives and GMB process set boundaries around the evidence utilized in the process. And finally, in the fourth section, we address concerns over representation by exploring which stakeholders were accounted for within the GMB process.

### SDM as a knowledge translation tool

From a KT perspective, the problem of interest is often specified in advance. From this starting point, SDM can provide decision-makers with ways to apply data developed by researchers, but also, through its participatory engagement via GMB activities, clarify for decision-makers where key informational gaps might lie and how to fill them through research efforts for those pre-defined problems. We identified two ways in which this occurs: (1) SDM can serve as a scoping/mapping tool, and (2) assist with gap-identification.

#### Scoping problems and solutions

During the GMB workshops, researchers provided evidence from the broader UO project to guide participants in the development of a preliminary CLD presenting broader factors influencing TB care systems. This diagram was then used to reflect on areas for intervention to improve TB IPC. One key way this enabled the application of useful evidence was by providing a framework for understanding how information fits into the problems and solutions being discussed. For example, as one policymaker explained:

*I think it’s [SDM] a good tool because it throws all the elements in the system, you know, because they are things that … kind of human resource dependent, and things that are dependent on management or maybe enforcement of policies that exist. But the things that … kind of related to patients themselves … the things that relate to more of infrastructure and logistics, that should be there in our facility. So I feel it kind of dissects it so that you can see what is the gap or what is not working well, so that at least your intervention can be kind of focused, and kind of helps to see where you can get more fields. Because it doesn’t help how to implement something that’s so costly, that will move maybe the needle by 2%. So you rather … allocate your effort and your resources knowing that if I do this, and they do this correctly, at least it can help me in kind of improving so much. (WKSHP 2, Interview 1-04, Policymaker)*

This excerpt reveals the nature of the ‘messy’ problem of TB IPC in South Africa, and one gets a sense of the many sectors and resources required to address the issue. As a KT tool, SDM helped to expose the disconnect between problems, solutions and current resource allocation, as well as providing an opportunity to ‘see’ the issue more clearly with all component pieces placed together, using the visuals and data provided. Similarly, another policymaker stated:

*[SDM is] like a scoping project as to where to source your various sources of information. (WKSHP 2, Interview 1-01, Policymaker)*

While SDM primarily serves to address strategic problems, it could also help to highlight where stakeholders can *source* the information they require to solve the specific problems discussed. Additionally, it can provide information from a research team, while also providing a way to map out data or informational needs, and potentially helping to think through where sources of useful data might be found to solve the problem.

#### Gap identification

In addition to helping identify sources of information, the process of mapping out the complexities of systems was also seen to help identify policy-relevant information gaps. For example, in one interview, a practitioner discussed the discrepancy between guidelines (and the research that informs these guidelines)—and what happens ‘on the ground’ within clinical settings. They explained:

*I really do wish that [the] gap between policymakers and people who think that they know what is in the best interest of a facility and for clinicians and patients … could be addressed. (WKSHP 2, Interview 2-01, Practitioner)*

The GMB workshops provide an opportunity for stakeholders to raise their concerns about gaps such as these and to link them to evidence-informed solutions. Several of the workshop participants also suggested that the SDM process was helpful in identifying lacunae in information by hearing other stakeholders’ viewpoints. One policymaker noted:

*What [GMB] does, is it gets around the table … [things] that you didn’t even consider … it takes you on a journey or process, where it opens up other doors that normally we [policymakers] [would] just walk past. (WKSHP 2, Interview 1-05, Policymaker)*

The GMB workshops were able to facilitate communication and engagement between participants, but created room to feed back evidence needs to researchers, creating a dynamic, iterative space for information-sharing. One practitioner, for instance, stated that ‘community engagement’ was missing and ‘essential’ to add to the model, which could then be addressed by researchers almost immediately (WKSHP 2, Main room session, Practitioner).

Overall, the SDM process served KT functions in a number of ways. Firstly, system complexities can be visually mapped providing a simple, broad representation of the various components that influence complex issues, generating shared understanding between different stakeholders about where pieces of data would fit into their needs. Secondly, it identified gaps in research knowledge and provided decision-makers the opportunity to feedback thoughts on those gaps or evidence needs to researchers. Lastly, it can provide a mechanism for the direct provision of research results to decision-makers, but also help to identify where information to address problems can be sourced.

### Programmatic tasks, goals and relevant evidence

In contrast to the KT approach which starts from a goal of getting uptake of previously gathered data and evidence, a programmatic perspective starts from the recognition that decision-makers may have multiple tasks and goals, each of which may have implications for which evidence is considered policy-relevant. We thus also investigated which programmatic goals the SDM appeared to provide useful information on from the perspective of participants. Within our interviews, we specifically asked several questions to allow policymakers and practitioners to identify their various roles and to reflect on evidence in relation to them.

Participants held multiple roles and carried out a variety of different tasks, however most were involved in some form of programme management. As one policymaker explained, their focus was to:

*prevent or control the spread of TB, make sure those that have [TB] are on treatment and … make sure that those [who] don’t have it, don’t get it, especially in our health facilities. (WKSHP 2, Interview 1-04, Policymaker)*

For both policymaker and practitioner categories, programme management included tasks related to ‘facility management or clinical governance’, the provision of training and the organization of human resources, in particular ‘recruitment and selection … induction and orientation’ activities (WKSHP 2, Interview 2-01, Practitioner). Programme management also involved budget management, that is:

*[going] over specific percentages for the month then [projecting] an overspending. If we are not overspending, we can project an underspending which means we can push money from one line item to another line item. (WKSHP 2, Interview 2-01, Practitioner)*

Other participants were tasked with focused monitoring and evaluation activities. For one policymaker, this meant coordinating the introduction of an integrated information system which aims to ‘improve patient management and care, clinical governance, as well as improve data quality’ (WKSHP 2, Interview 1-05, Policymaker). This led to quite specific conceptions of what relevant data or evidence was. For example, one practitioner utilized a tool called the Ideal Clinic checklist for their monitoring and evaluation task (Department of Health, 2020). As a web-based application, this checklist allows managers at all levels to monitor the progress of their clinics. For this individual, the information required to assess clinical performance was provided by the framework, and ‘relevant’ information which included information such as the number of medicines and supplies available was then obtained to check against these checklist items. Many participants interviewed were asked to explain more explicitly what makes evidence policy- relevant. They spoke of characteristics of data that were required to achieve their specific tasks. Local data, or evidence generated from within South Africa, were seen to be relevant evidence for both policymakers and practitioners. These data were understood to be distinct from global data—such as the evidence sourced from international guidelines on TB IPC—and were also seen to be essential in making sure international and national policies were adapted to the provincial or district context. For those undertaking budget management tasks, local data were said to be obtained through ‘local costing data’, ‘cost-effectiveness evaluations’, investment cases and ‘local stakeholder data’—all of which could ‘inform future budget planning’ (WKSHP 2, Interview 1-01, Policymaker). For others undertaking monitoring and evaluation activities, ‘survey acceptance’, ‘utilization patterns’, analytics from information systems and patient- and population-based outcomes were also seen to be important data required to ascertain a broad, clear picture of ‘local’ needs (WKSHP 2, Interview 1-01, Policymaker).

This variety of tasks and goals of participants, and the relevant evidence seen to assist in achieving them, has implications when reflecting on the role of the SDM process. Two tasks that SDM was seen to be able to facilitate were monitoring and evaluation and programme management. As one policymaker involved in conducting monitoring and evaluation activities explained, ‘If you use system modelling, you identify challenges, and then it also guides you [on] how to address each of those challenges’ (WKSHP 2, Interview 1-02, Policymaker).

Another policymaker engaged in monitoring and evaluation echoed this, saying that SDM could help ‘analyse the problem’, find ‘new ways of doing things’ and ‘fill gaps and possibilities’ in implementation (WKSHP 2, Interview 2-03, Practitioner). Similarly, speaking with reference to how SDM can assist with programme management, one policymaker said that ‘[SDM] assists us to plan ahead’ and explained that SDM, unlike other tools that might utilize a more linear approach, allows for ‘cyclical planning’ (WKSHP 2, Interview 1-02, Policymaker).

Despite the widespread perception among participants that SDM was able to facilitate a variety of different tasks, some felt that it was not always the most effective approach for the types of decision-making they had to undertake. One reason for this was that the SDM process was not seen as being especially practical for the context. As one practitioner explained:

*I think there were some things that are a little bit far-fetched and very theoretical, that I think wouldn’t work in our context. (WKSHP 2, Interview 2-02, Practitioner)*

Another practitioner touched on the issue of SDM’s incompatibility with this ‘context’ in further detail:

*I think your biggest challenge is to get people to buy in … Not everyone, including myself, [was] exposed to systems thinking in school … So, the one challenge is everyone is not exposed to … thinking in that way. Two, not everyone understands it. I think number three, within the systems model thinking, it’s heavily linked to digital … like utilizing of a computer … and a lot of our staff are not computer literate. (WKSHP 2, Interview 2-01, Practitioner)*

Others echoed this sentiment by saying that it was ‘not user friendly’ (WKSHP 2, Interview 1-01, Policymaker) and that it would require more training workshops to get all members involved in the GMB workshops on the same page (WKSHP 2, Interview 1-04, Policymaker). Thus, from a programmatic perspective, SDM was seen as providing potentially useful information for some but not all practitioners’ goals, yet it might not provide the form of evidence most conducive to the teams involved.

### Research objectives as setting the boundaries for evidence use

The third approach to reflect on evidence use is to recognize that the process of evidence utilization itself can serve to construct or formalize the bounds of the policy problem itself, establish conceptual understandings and delineate particular solution sets for consideration.

One way to see this unfold is in how the SDM language used during the GMB workshops could lead to participants adopting certain terminology to think through problems and solutions. For example, one practitioner stated:

*[SDM] would be a tool for me to build specific processes and systems. [If] I’ve volunteered to use the systems dynamics tool, then I will utilize it to build the specific processes and systems and flows to achieve what has to be achieved … And then taking from that, everything else will then flow and become the system that we envision … you know, we built, for example, double loop systems and a double loop system is … if a client complained about receiving expired medication, we want to do a root cause analysis as part of the whole system dynamics … and then once we understand the problem, we innovate, and it’s called double loop because now we understand the problem, we’ve innovated and we can prevent it from happening again. (WKSHP 2, Interview 2-01, Practitioner)*

Here, the participant uses SDM-type language such as ‘processes’, ‘flows’ and ‘double loop systems’ to think through how SDM could be an integrated process embedded in the organization and functioning of the health facility that they manage. Providing knowledge of the SDM process also gives people a new framework with which to see how existing systems function—or where the gaps are—in a novel way.

However, while participants in the GMB workshops brought their own understandings of the problem of TB transmission, as well as of how they thought it could be addressed, the objectives of the research project itself played an important role in shaping how the problem was framed and solutions constructed. For instance, the second GMB workshop presented information on three central areas where specific dynamics were found to affect nosocomial transmission: (1) actual drivers of transmission at the clinic level; (2) the clinic working environment; and (3) the broader policy environment which shapes the clinic environment and the types of programmes implemented at the clinic level. The workshop also included the presentation of results from the mathematical modelling of various interventions (clinic and community), and SDM was presented as being useful for costing in order to facilitate a priority-setting exercise to: (1) allocate scarce resources efficiently to maximize health benefits; (2) adapt to local needs (both prices and epidemiological characteristics); and (3) rank and optimize across multiple interventions.

These elements, however, represent only a sub-sample of all the possible pieces of information that might be considered policy-relevant evidence, depending on which tasks, goals and construction of the problem are being considered. For example, Ultraviolet Germicidal Irradiation (UVGI) was one intervention presented by researchers and discussed during the second GMB workshop. However, one participant acknowledged that they did not have enough knowledge about UVGI to fully comprehend its benefits:

*For me, the discussion around UV especially in the context of what other benefits it brings … my knowledge on this needs to improve … besides TB what other possible advantages [does] it bring? (WKSHP 2, Main room session)*

This example of the limitations of knowledge regarding the interventions developed during the SDM process, informed by evidence generated by the researchers, may have implications for which ideas and approaches to infection control became realized in the discussions.

### Participation and representation

Finally, once there is recognition that different policy needs exist, and that problems and policy-relevant evidence can be constructed in different ways, it follows that it may be particularly important to consider which groups or individuals are involved in the processes leading to such constructions—to consider if the values or priorities being embedded into these constructions are representative of the population or the wider community for whom the policies are meant to serve.

The SDM process enables various stakeholders to participate in a process of multi-disciplinary teamwork during GMB workshops to identify problems and solutions. Indeed, the inclusion of multiple stakeholder perspectives was emphasized by some of the interviewees as being a major benefit of using SDM for policymaking. As one participant explained:

*What interested me with that modelling [GMB exercise], as complicated as it is … is that it includes many stakeholders. Because, for example, every workshop, they make sure that government is involved, labour or professional associations are involved, victims or people who were infected were part of that … representatives from communities were involved in that … so they took all the inputs of everyone and [made] sure that [the] model tried to accommodate each level of understanding. (WKSHP 2, Interview 2-03, Practitioner)*

This multidisciplinary involvement during the GMB workshops was seen to be beneficial, not only because various stakeholder views were represented, but also because it enabled some participants to feel as although they were working together towards a common objective:

*[The] benefit of [SDM] is that we need to work as a team. We can’t work alone, because as a team … you need engineers, we need a patient advocate … Because all of us in health … are focusing on health … whether you are a healthcare worker, an information person, an infrastructure person … We look at those dynamics … it assists us to work as a team and achieve our objective as a team, together. (WKSHP 2, Interview 1-02, Policymaker)*

While GMB may have assisted participants to ‘work as a team’ towards a common objective, there could be differences in perspectives shaping perceptions of relevant evidence. For instance, one policymaker spoke of the ‘policy perspective’ when discussing the application of relevant evidence to refer to all the pieces of information that might inform policy decision-making (WKSHP 2, Interview 1-01, Policymaker). Evidence in the form of ‘utilization patterns, cost-effectiveness and survey acceptance’ all seemed to illustrate a ‘top-down’, ‘government perspective’ to the application of relevant evidence (WKSHP 2, Interview 1-01, Policymaker). Other policymakers also referred to the need for ‘the most recent evidence of models that work’ (WKSHP 2, Interview 1-04, Policymaker), training manuals informed from evidence ‘based in different countries’ (WKSHP 2, Interview 2-03, Practitioner) and evidence generated from monitoring and evaluations checklists such as the Ideal Clinic checklist (WKSHP 2, Interview 1-02, Policymaker).

This perspective contrasted with some practitioners, however, who referred to the application of relevant evidence that was more facility-specific. For example, as one practitioner explained:

*facilities are now capturing their own TB data … immediate data that’s at the facility level and subdistrict level makes it a bit easier for us to plan. We have facilities we want to roll out our TB services to, so obviously we need to take into account what the community level of TB infection is … and depending on that, we … will staff appropriately, get things in order and then protect our staff as well. (WKSHP 2, Interview 2-02, Practitioner)*

A similar sentiment was expressed by another practitioner who felt relevant evidence was ‘good-quality data’ that could be used to develop ‘a strategic plan for the facility, as well as an operational plan’ (WKSHP 2, Interview 2-01, Practitioner). From both accounts, we see the distinction in the types of evidence deemed relevant by policymakers and practitioners. As such, the idea that SDM can facilitate gap identification and that it occurs through a process of multidisciplinary teamwork could be made more effective by providing standard information to all stakeholders preceding GMB activities to ensure all participants are on a level playing field.

## Discussion

As a methodology, SDM is progressively being used in policy spaces as a framework to delineate problems and plan their associated solutions as well as to identify pieces of information deemed policy-relevant. In doing so, there are a number of implications in relation to key considerations raised by scholars concerned with the use of evidence for policy-making more broadly. While many authors have previously recognized that the GMB approach serves as an effective communication tool and develops a shared understanding of ‘messy’ problems (Vennix, 1995; Andersen *et al.*, 1998; Black, 2013; Luna-Reyes *et al.*, 2019), our study further explores how it may also serve as a KT tool by assisting with mapping/scoping problems and solutions, sourcing information and identifying gaps in knowledge. Similar to Kitson *et al*. (2018), who offer a ‘multi-dimensional, iterative and flexible’ approach to understanding KT using complexity and network concepts, we found GMB to facilitate KT between researchers, practitioners and policymakers where ‘translation gaps’—such as those between policies and what happens ‘on the ground’—were found to occur in a dynamic manner.

However, we also found context-specific challenges in relation to specific features of where it was being applied. For instance, due to the software used to generate CLDs, some respondents were not convinced that it could be used for routine decision-making, particularly within clinical settings. Similarly, not all participants had the same level of SDM knowledge nor of TB-related interventions. To account for difference in levels of knowledge, UO researchers conducted separate workshops asking slightly different questions—practitioners identified where risk of transmission was higher using a plan of the clinic—while policymakers mapped the history of TB IPC policy. This ensured that workshops were targeted to participants’ type and level of knowledge. However, it also meant that some participants may not have had enough relevant knowledge to fully contribute to all discussions and debates.

This observation points to the importance of participation and representation within the process. Hovmand (2014, p. 10) recognizes that there are ‘different views of what constitutes participation which may also have political implications’. In our case study, ‘participation’ was seen to consist of a multi-disciplinary group of stakeholders and thus was ‘representative’ of the various stakeholders’ views, tasks and goals. Participants therefore felt they were working collaboratively towards a ‘common goal’. When examining the relevance of evidence in particular as it pertains to our two participant categories, we found distinct understandings of relevance that a programmatic approach would highlight by exploring what would help achieve their particular tasks and goals. For example, policymakers felt that the ‘government perspective’ was a priority, whilst practitioners were more concerned that ‘facility-specific’ interests were met. Working collaboratively, however, the meaning of the TB IPC problem gravitated towards a ‘common goal’ re-emphasizing Zimmermann and Black’s (2015) claim that the use of participatory methods can change meaning—or in our case could reshape perceptions of which evidence was seen as relevant. Hosseinichimeh *et al*. (2019) suggest that a power-by-interest grid be completed by the modelling team to identify GMB participants to systematically determine a wider range of stakeholders; however, a way to navigate potential bias in attention to particular issues or concerns in the views that emerge during GMB workshops is still required. Despite this, as in other literature (Luna-Reyes *et al.*, 2019; Martin, 2021; Morais *et al.*, 2021), we found that the GMB workshops enabled discussion and debate between a variety of different stakeholders, and in turn, facilitated a greater understanding of problems identified and their associated solutions.

Of course, there is a recognition here that the evidence being fed into discussions by researchers may have led to a focus on (or construction of) specific problems and solutions rather than ones that could have potentially been seen if an alternative approach were taken (for instance, starting with policymakers’ or practitioners’ experiences and knowledge, and working from there). This is not to judge in one way or another which is a better approach. Pre-defined categories and problem constructions allow clarity and prioritization of certain variables and considerations, which the SDM process can potentially serve. But these results help to provide an example of how particular constructions subsequently direct problem definition and evidentiary considerations in one or another direction—and how they could exclude alternatives as well.

## Conclusion

The growth in the application of planning tools deriving from complexity science to inform health and broader public policy decision-making is likely to continue. Yet with the health sector’s well-established embrace of the idea that policies should be informed by evidence, this study has aimed to explore how the process of undertaking an SDM process can function in relation to evidence provision in a specific health system context. This is done in recognition of a range of critical considerations that policy scholars have raised in the policy sciences in relation to evidence utilization for public policy.

In our case, SDM was clearly a useful tool and process to help facilitate the understanding and utilization of research evidence in many ways. Yet as the conceptual discussion and analysis point out, there are other considerations around evidence use beyond KT that can also be considered. A programmatic approach to evidence use starts by reflecting on the multiple goals and tasks pursued by decision-makers, to consider which evidence is most relevant in relation to those. Furthermore, constructivist scholars raise questions about how the use of particular forms of evidence can serve to frame and construct policy problems and their solutions; while those concerned with the propitiation of competing social values raise questions about representation amongst groups involved in evidence use. Each of these lenses can provide insights into ways that the SDM process relates to evidence use, and the political nature of that use in practice. Such critical perspectives will be important to consider as planning tools such as SDM and GMB continue to increase in popularity and in their application as a policy-informing tool—both in the health sector and beyond.

## Reflexivity statement

### Regional location and experience

The research team was acutely aware of the need to include both local authors and authors that have a good awareness of the wider social and political context of South Africa when writing this paper. SP, the first author, has been based in South Africa for the past nine years. She is a Registered Social Worker and is currently conducting doctoral research in clinical settings similar to those discussed in this paper. She therefore has sound knowledge of and experience working in this setting. AG is based in London, but most of her research has primarily been based in South Africa since 1997, where her work has focused on the development and evaluation of interventions to reduce HIV-related illness and death, particularly HIV-related TB. JP, AV, FB and KD are based in the UK. KK is based in Belgium. This range of regional location provided a good balance of perspectives in authoring this paper.

### The role of the research team as collaborators in knowledge production

Within the broader Umoya Omuhle project, there was a strong commitment to work collaboratively with both local and international researchers in the collection, analysis, interpretations and reporting of the data. In our specific study, the team members most closely involved in data collection and analysis (SP and JP) met frequently to reflect on the progress of fieldwork and analysis. At this stage, input was sought from the other members of the research team who had extensive experience of qualitative research and a broad knowledge of health policy, evidence-based research, SDM and TB IPC. Throughout the drafting of this paper, all co-authors provided input based on their experience and knowledge of the subject matter.

## Sex and seniority

Within the context of our study, the members of the research team who took part in the authorship of this paper consisted of six women and one man of various levels of seniority and experience. SP, the first author of this manuscript, is an early career researcher and current PhD candidate in Public Health. Close assistance was provided by JP, Associate Professor of Global Health Policy, throughout all stages of research. Further guidance was provided by AG (physician and Professor of International Health), KK (Professor of Equity and Health), FB (Assistant Professor in Health Economics), AV (Professor of Health Economics) and KD (an experienced SDM researcher and modeller).
